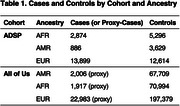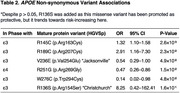# Non‐synonynous *APOE* variants association in diverse ancestries

**DOI:** 10.1002/alz70861_108322

**Published:** 2025-12-23

**Authors:** Yann Le Guen, Andrés Peña‐Tauber, Michael D. Greicius

**Affiliations:** ^1^ Quantitative Sciences Unit, Department of Medicine, Stanford University School of Medicine, Stanford, CA USA; ^2^ Department of Neurology and Neurological Sciences, Stanford University School of Medicine, Stanford, CA USA

## Abstract

**Background:**

Despite decades of research, mechanisms linking *APOE* to Alzheimer’s disease (AD) remain poorly understood. Recent studies have highlighted the importance of population diversity in uncovering novel risk and protective variants at the *APOE* locus. Here, we investigate non‐synonymous *APOE* variants in individuals of African (AFR), Admixed‐American/Native American (AMR), and European (EUR) ancestries to elucidate how additional missense changes modulate AD risk beyond the ε2 and ε4 alleles.

**Method:**

We tested associations of *APOE* non‐synonymous variants with minor allele count ≥10 in two large cohorts: the Alzheimer’s Disease Sequencing Project (ADSP; release ng00067.v12: 36K WGS, 20K WES) and All of Us (414K WGS). Logistic regression models adjusted for sex, *APOE*‐ε2 and *APOE*‐ε4 dosages, and 10 (ADSP) or 20 (All of Us) genetic principal components. Ancestry assignments were based on proximity to 1000 Genomes reference clusters. Cases and controls per ancestry are detailed in Table 1. Cohort‐specific effect estimates were meta‐analyzed with the inverse variance weighted method, with All of Us effect sizes and standard errors doubled to account for the use of proxy‐cases (ICD‐10 G30 diagnosis or first‐degree family history).

**Result:**

A subset of missense variants reached nominal significance (Table 2). Two novel variants enriched in Native American–admixture (in phase with ε3)—W276C (OR=0.14, 95%CI 0.02–0.98; *p* =4.8×10⁻²) and R189C (OR=2.91, 95%CI 1.16–7.30; *p* =2.3×10⁻²)—were associated with decreased and increased AD risk, respectively. We also replicated known associations: R145C (OR=1.32, 95%CI 1.10–1.58; *p* =2.6×10⁻³), V236E “Jacksonville” (OR=0.54, 95%CI 0.29–1.00; *p* =4.9×10⁻²), and R251G (OR=0.47, 95%CI 0.26–0.86; *p* =1.5×10⁻²). The Christchurch variant R136S trended toward risk elevation (OR=8.25, 95%CI 0.42–162.41; *p* =1.6×10⁻¹).

**Conclusion:**

We identified two novel Native American–specific missense variants at *APOE*—W294C and R189C—that modulate AD risk and confirmed established protective and risk‐increasing alleles. Of note, the mature APOE3 amino‐acid chain (excluding the 18‐residue signal peptide) contains only one cysteine at position 112, underscoring the protein’s sensitivity to cysteine substitutions. The disproportionate impact of these thiol‐forming changes—akin to those defining ε2 (R158C) and ε4 (C112R)—highlights a critical role for cysteine chemistry in APOE function and AD pathogenesis. Functional characterization of W294C and R189C may reveal novel therapeutic pathways.